# Neutering Dogs: Effects on Joint Disorders and Cancers in Golden Retrievers

**DOI:** 10.1371/journal.pone.0055937

**Published:** 2013-02-13

**Authors:** Gretel Torres de la Riva, Benjamin L. Hart, Thomas B. Farver, Anita M. Oberbauer, Locksley L. McV. Messam, Neil Willits, Lynette A. Hart

**Affiliations:** 1 Department of Population Health and Reproduction, School of Veterinary Medicine, University of California-Davis, Davis, California, United States of America,; 2 Department of Anatomy, Physiology and Cell Biology, School of Veterinary Medicine, University of California-Davis, Davis, California, United States of America; 3 Department of Animal Science, College of Agriculture and Environmental Sciences, University of California-Davis, Davis, California, United States of America; 4 Department of Public Health Sciences, School of Medicine, University of California-Davis, Davis, California, United States of America; 5 Statistics Laboratory, Department of Statistics, University of California-Davis, Davis, California, United States of America; Van Andel Institute, United States of America

## Abstract

In contrast to European countries, the overwhelming majority of dogs in the U.S. are neutered (including spaying), usually done before one year of age. Given the importance of gonadal hormones in growth and development, this cultural contrast invites an analysis of the multiple organ systems that may be adversely affected by neutering. Using a single breed-specific dataset, the objective was to examine the variables of gender and age at the time of neutering versus leaving dogs gonadally intact, on all diseases occurring with sufficient frequency for statistical analyses. Given its popularity and vulnerability to various cancers and joint disorders, the Golden Retriever was chosen for this study. Veterinary hospital records of 759 client-owned, intact and neutered female and male dogs, 1–8 years old, were examined for diagnoses of hip dysplasia (HD), cranial cruciate ligament tear (CCL), lymphosarcoma (LSA), hemangiosarcoma (HSA), and mast cell tumor (MCT). Patients were classified as intact, or neutered early (<12 mo) or late (≥12 mo). Statistical analyses involved survival analyses and incidence rate comparisons. Outcomes at the 5 percent level of significance are reported. Of early-neutered males, 10 percent were diagnosed with HD, double the occurrence in intact males. There were no cases of CCL diagnosed in intact males or females, but in early-neutered males and females the occurrences were 5 percent and 8 percent, respectively. Almost 10 percent of early-neutered males were diagnosed with LSA, 3 times more than intact males. The percentage of HSA cases in late-neutered females (about 8 percent) was 4 times more than intact and early-neutered females. There were no cases of MCT in intact females, but the occurrence was nearly 6 percent in late-neutered females. The results have health implications for Golden Retriever companion and service dogs, and for oncologists using dogs as models of cancers that occur in humans.

## Introduction

The overwhelming majority of companion dogs maintained in the U.S. are spayed or castrated (both referred to herein as neutered) [1]. Increasingly in the U.S. neutering is being performed early, demarcated in the present study as prior to one year of age. The impetus for this widespread practice is presumably pet population control, and is generally considered responsible pet ownership. However, this societal practice in the U.S. contrasts with the general attitudes in many European countries, where neutering is commonly avoided and not generally promoted by animal health authorities. For example, a study of 461 dogs in Sweden reported that 99 percent of the dogs were gonadally intact [2], and an intact rate of 57 percent was reported in a Hungarian study [3]. In the United Kingdom, a 46 percent intact rate was reported [4].

In the last decade, studies have pointed to some of the adverse effects of neutering in dogs on several health parameters by looking at one disease syndrome in one breed or in pooling data from several breeds. With regard to cancers, a study on osteosarcoma (OSA) in several breeds found a 2-fold increase in occurrence in neutered dogs relative to intact dogs [5]. Another study on OSA, to explore the use of Rottweilers as a model for OSA in humans, found that neutering prior to 1 year of age was associated with an increased occurrence of OSA; 3–4 times that of intacts [6].

Hemangiosarcoma is a cancer that is affected by neutering in females. A study of cardiac tumors in dogs found that cardiac HSA for spayed females was greater than 4 times that of intact females [7]. A study on splenic HSA found the spayed females had more than 2 times the risk of developing this tumor as intact females [8]. Neither of these studies separated early- versus late-spayed females with regard to increased risk, and neither focused on just one breed. A study on the epidemiology of LSA (lymphoma) in dogs, for comparison with human lymphoma, found that intact females had a significantly lower risk of developing this cancer than neutered females or neutered males or intact males [9]. Another cancer of concern is prostate cancer, which occurs in neutered males about four times as frequently as in intact males [10]. A study on cutaneous mast cell tumors (MCT) in several dog breeds, including the Golden Retriever, examined risk factors such as breed, size, and neuter status. Although early versus late neutering was not considered, the results showed a significant increase in frequency of MCT in neutered females; four times greater than that of intact females [11].

In contrast to the rather strong evidence for neutering males and/or females as a risk factor for OSA, HSA, LSA, MCT, and prostate cancer, evidence for neutering as protection against a dog acquiring one or more cancers is weak. The most frequently mentioned is mammary cancer (MC) [12]. However, a recent systematic review of published work on neutering and mammary tumors found the evidence that neutering reduces the risk of mammary neoplasia to be weak, at best [13].

With regard to joint disorders affected by neutering, one study documents a 3-fold increase in excessive tibial plateau angle – a known risk factor for development of CCL – in large dogs [14]. A paper on CCL found that, across all breeds, neutered males and females were 2 to 3 times more likely than intact dogs to have this disorder [15]. In this study, with sexes combined, neutering significantly increased the likelihood of HD by 17 percent over that of intact dogs.

Given the widespread practice of neutering in the U.S., especially with public campaigns promoting early neutering, and the contrast with neutering practices in other developed countries, the objective of this project was to retrospectively examine the effects of neutering on the risks of several diseases in the same breed, distinguishing between males and females and early or late neutering versus remaining intact using a single hospital database. Because neutering can be expected to disrupt the normal physiological developmental role of gonadal hormones on multiple organ systems, one can envision the occurrence of disease syndromes, including those listed below, to possibly be affected by neutering as a function of gender and the age at which neutering is performed. The study focused on the Golden Retriever, which is one of the most popular breeds in the U.S. and Europe. In this breed, HD, CCL, LSA, HSA, MCT, OSA, and elbow dysplasia (ED) are listed as being of particular concern [16].

## Methods

### Ethics Statement

No animal care and use committee approval was required because, in conformity with campus policy, the only data used were from retrospective veterinary hospital records. Upon approval, faculty from the University of California, Davis (UCD), School of Veterinary Medicine, are allowed restricted use of the record system for research purposes. The final dataset used for statistical analyses is available to qualified investigators, upon request, from the corresponding author.

### Data Collection

The dataset used in this study was obtained from the computerized hospital record system (Veterinary Medical and Administrative Computer System) of the Veterinary Medical Teaching Hospital (VMTH) at UCD. The subjects included were gonadally intact and neutered female and male Golden Retrievers, 1 to 8 years of age and admitted to the hospital between January 1, 2000 and December 31, 2009. Data from patients less than 12 months of age and 9 years or older were not considered. Additional inclusion criteria were requirements for complete information on date of birth, date of neutering (if neutered) and date of diagnosis (or onset) of the joint disorder or cancer. Patients were classified as intact or neutered; the neutering was sub-classified as “early” if done before 12 months of age and “late” if done at 12 months of age or older. For all neutered patients, the neuter status at the time of each visit was reviewed to ensure that neutering occurred prior to onset of the first clinical signs or diagnosis of any disease of interest.

While the study set out to estimate incidence rates related to age at the time of neutering, patients were diagnosed at different ages and with differing durations of the disease as well as varying years of exposure to the effects of gonadal hormone removal. For those intact, early-neutered and late-neutered dogs diagnosed with a disease, the age of diagnosis was recorded. Follow-up times were recorded for each patient and determined by age of the dog at the initial clinical signs or diagnosis, minus the age of the dog when first included in the study. For dogs with no disease, follow-up times were the age at the last visit to the VMTH minus the age when the dog was first included in the study.

With the goal of obtaining a sample size sufficiently large for statistical analysis, the database records were initially screened using disease-related keywords to evaluate the frequency of occurrence of HD, CCL, HSA, LSA, MCT, ED, OSA, and MC. Extensive reviews of patient records were then performed for specific evidence and information on each joint disorder or cancer for every patient included in the study. Only diseases with at least 15 cases found using this screening were included in the study.

For all patients where age at time of neutering was not available in the record, an effort was made to obtain the information by telephone from the referring veterinarian. At the same time, age of onset of the disease in question was also sought. If the information was not available from the referring veterinarian, an attempt was made to reach the dog owner for this information. In order to optimize success in obtaining information, these efforts were focused on patients born in 2000, or later, and that were admitted to the VMTH between January 1, 2005 and December 31, 2009.

Table 1 defines the categories of diagnoses based on information in the record of each case. A patient was considered as having a disease of interest if the diagnosis was made at the VMTH or by a referring veterinarian and later confirmed at the VMTH. Patients clinically diagnosed with HD and/or CCL presented with clinical signs such as difficulty standing up, lameness, or joint pain; diagnosis was confirmed with radiographic evidence and/or orthopedic physical examination. Clinical diagnoses of the various cancers were accompanied by clinical signs such as enlarged lymph nodes, lumps on the skin or presence of masses, and confirmed by imaging, appropriate blood cell analyses, chemical panels, histopathology and cytology. When a diagnosis was suspected based on clinical signs, but the diagnostic tests were inconclusive or not done, telephone calls were made to referring veterinarians and owners to confirm the diagnosis. Lacking a conclusive confirmation, the case was excluded from the analysis for that specific joint disorder or cancer. Finally, body condition scores (BCS), ranging from 1 to 9 and obtained from the patient records (when available) were taken into account because BCS, as an indication of weight on the joints, is considered to play a role in the onset of these joint disorders [17], [18]. Also, neutering has been implicated in an increase in body weight, especially as indicated by body condition score [18].

**Table 1:** Categories used in determining diagnosis for joint disorders and cancers of interest in Golden Retrievers (1–8 years old) admitted to the Veterinary Medical Hospital, University of California, Davis, from 2000–2009.

Classification	Definition
No disease	No evidence of a joint disorder or cancer of interest in the medical records
VMTH	Diagnosed at the VMTH
Referring Veterinarian/VMTH	Diagnosed by referring veterinarian and confirmed at the VMTH
Referring Veterinarian	Diagnosed by referring veterinarian but no diagnostic tests done at the VMTH
Suspected	Diagnosis was suspected based on clinical signs, but diagnostic tests were inconclusive or not done, telephone calls were then made to referring veterinarians and owners to confirm diagnosis, unconfirmed cases were excluded from analysis for the suspected joint disorder or cancer
Invalid	Diagnosed prior to January 2000 or after December 2009 and before 1 year of age or 9 years of age and older were excluded from analysis for the specific joint disorder or cancer

### Statistical Analyses

Kaplan-Meier survival analysis (K-M) [19] was used to estimate survival curves for each disease and neuter status by gender, and then log-rank and generalized Wilcoxon tests were used for post hoc comparisons between a set of two curves and thus to evaluate differences in occurrence of the diseases of interest in each comparison group. Incidence rate estimates (IR) [20] were used to evaluate the rates of disease onset using time-at-risk of the disease, in this case, dog-years at risk. Time-at-risk for a disease is the duration of time each patient was observed prior to the disease occurrence. For late-neutered dogs, time-at-risk prior to neutering was used in the IR estimation for intact dogs and time observed after neutering was used in the IR estimate for late-neutered dogs. For each disease, rate ratios (RR) and their corresponding 95 percent confidence intervals (95% CI) were used to compare the rates of acquiring each disease with regard to neuter status (i.e., intact vs. early neutered, intact vs. late-neutered, and early- vs. late-neutered). To examine the role of BCS in the development of HD and CCL, Cox proportional hazard (CPH) models were used, in which both BCS and age at the time of neutering were included as predictors. The resulting tests of the neutering effect are adjusted for differences in BCS among the groups. Statistical level of significance was set at the 5 percent level for all analyses.

## Results

**Figure 1:**
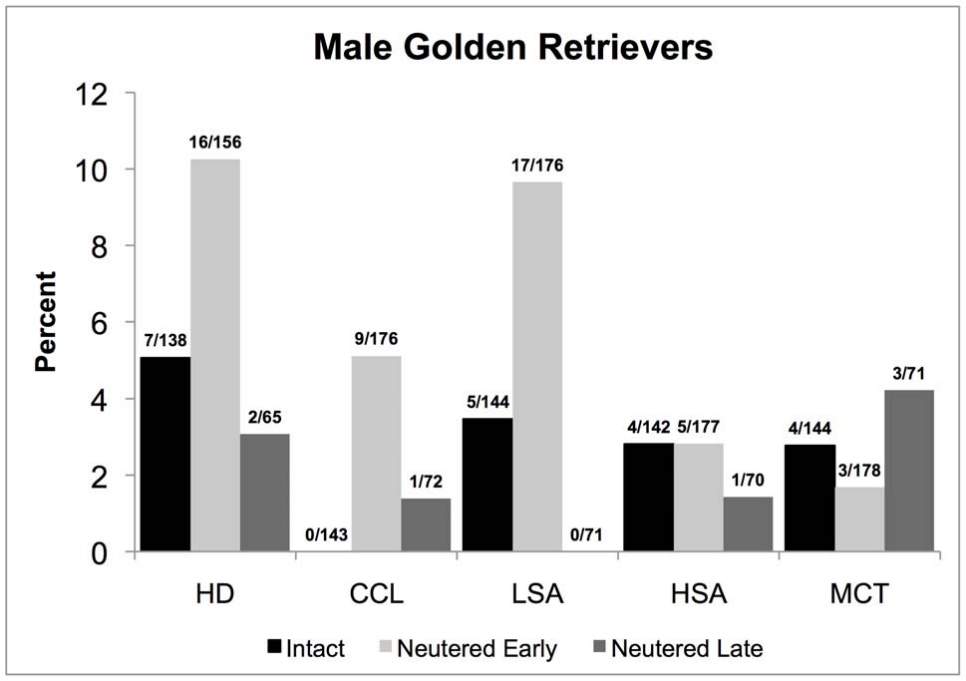
Percentages and number of cases over the total sample size for each neutering status group; intact and neutered early or late for male Golden Retrievers (1–8 years old) diagnosed with hip dysplasia (HD), cranial cruciate ligament tear (CCL), lymphosarcoma (LSA), hemangiosarcoma (HSA), and/or mast cell tumor (MCT) at the Veterinary Medical Teaching Hospital of the University of California, Davis, from 2000–2009. For HD and LSA, the differences between early-neutered and intact or late-neutered groups were statistically significant (K-M), as were differences for CCL between intact and early-neutered groups.

**Figure 2:**
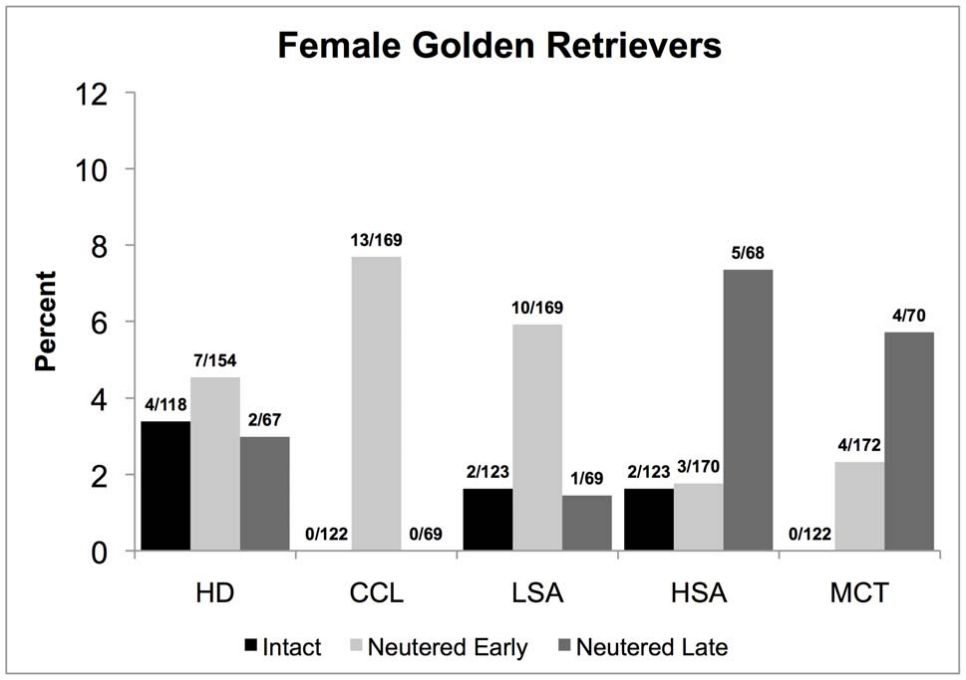
Percentages and number of cases over the total sample size for each neutering status group; intact and neutered early or late for female Golden Retrievers (1–8 years old) diagnosed with hip dysplasia (HD), cranial cruciate ligament tear (CCL), lymphosarcoma (LSA), hemangiosarcoma (HSA), and/or mast cell tumor (MCT) at the Veterinary Medical Teaching Hospital of the University of California, Davis, from 2000–2009. For CCL the difference between intact and early-neutered was statistically significant (K-M). For HSA, the differences between early and late-neutered and intact and late-neutered groups were statistically significant (RR), as were differences for MCT between early and late-neutered groups. A similar statistical comparison for late neutering and intact groups was not possible for MCT because there were 0 cases in the intact group.

Table 2 presents the sample size for each joint disorder or cancer of patients meeting all inclusion criteria, separately for males and females according to neuter status classification as to intact, early-neutered, and late-neutered. The number of subjects available for analyses of each disease varied because a patient could be excluded from the analyses for one disease, if for example, the diagnosis was made prior to one year of age or after 8 years, but would be included for analyses of all other diseases that may occur within the ages 1 to 8 years. A case could be considered as intact for one disease if onset was prior to neutering and as late-neutered for another disease that may have occurred after neutering. Meeting all inclusion criteria were 145 intact males, 178 early-neutered males, 72 late-neutered males, 122 intact females, 172 early-neutered females, and 70 late-neutered females (Table 2). The overall percentages of cases in the sample for the five diseases affected by early and/or late neutering considered for statistical analyses are presented in Figure 1 for males and in Figure 2 for females. Mean follow-up times for all the diseases of interest in intact, early-neutered and late-neutered dogs are listed in Table 3.

**Table 2:** Total sample sizes obtained for male and female Golden Retrievers (1–8 years old) admitted to the Veterinary Medical Hospital, University of California, Davis, from 2000–2009 according to neuter status classification: intact, early-neutered, and late-neutered.

Disease	Total	Intact	Neutered Early	Neutered Late
Males				
HD	359	138	156	65
CCL	391	143	176	72
LSA	391	144	176	71
HAS	389	142	177	70
MCT	393	144	178	71
Total*	395	145	178	72
Females				
HD	339	118	154	67
CCL	360	122	169	69
LSA	361	123**	169	69
HAS	361	123**	170	68
MCT	364	122	172	70
Total*	364	122	172	70

* Total number of dogs meeting all inclusion criteria.

** Includes patients that were diagnosed with a disease of interest prior to eventual late neutering.

**Table 3:** Mean follow-up times for male and female Golden Retrievers (1–8 years old) admitted to the Veterinary Medical Hospital, University of California, Davis, from 2000–2009 by disease status for each neuter category.

Disease	Intact	Early Neutered	Late Neutered
Males			
No Disease	2.12	3.16	1.77
HD	2.61	2.11	0.99
CCL	NA	3.37	NA
LSA	3.36	3.67	NA
MCT	3.45	3.53	2.98
HAS	3.05	4.57	NA
Females			
No Disease	1.48	2.48	1.40
HD	1.12	2.13	0.05
CCL	NA	3.16	NA
LSA	3.62	4.99	NA
MCT	5.70	4.44	2.28
HAS	5.37	2.70	3.23

NA =  Not applicable because there were no cases of the specific joint disorder or cancer in that neuter category.

As shown in Table 4, K-M survival analysis revealed that early neutering was associated with an increased occurrence of HD, CCL, and LSA. As shown in this table, comparisons of the IR analyses reveal that late neutering was associated with the subsequent occurrence of MCT and HSA in females. After the initial screening, ED, OSA, and MC occurred in such low numbers that statistical analyses were not feasible. MC was diagnosed in only two cases in the total number of 364 females, both in late-neutered females.

**Table 4:** Summary of some Kaplan-Meier post hoc comparisons using log-rank (LR) and generalized Wilcoxon (W) tests for male and female Golden Retrievers (1–8 years old) admitted to the Veterinary Medical Hospital, University of California, Davis, from 2000–2009.

Disease	Gender	Test type	*p* (early vs. intact)	*p* (early vs. late)	*p* (late vs. intact)
**Males**					
HD		LR	0.04	NS	NS
		W	0.01	0.04	NS
CCL		LR	0.003	0.02	NS
		W	0.004	0.01	NS
LSA		LR	0.01	0.002	NS
		W	0.04	0.01	NS
**Females**					
CCL		LR	0.001	0.001	NS
		W	0.006	0.004	NS

NS =  Statistically non significant.

### Hip Dysplasia

Perusal of Figure 1 and Table 4 reveals that HD in early-neutered males, affecting 10.3 percent, was more than double the proportion of intact males with the disorder, which was 5.1 percent, a significant difference (K-M: p<0.01). There was also a significant difference between early and late neutering in males (K-M: *p*<0.05). The mean ages of HD onset for intact, early-neutered, and late-neutered male dogs were 4.4, 3.6, and 4.7 years, respectively. No difference was found between early-neutered dogs with and without HD when compared with respect to their BCS, (means 6.1 and 5.7, respectively; CPH: p = 0.22). No other comparisons of HD occurrence were significant; HD was not increased in occurrence by early or later neutering in females (Figure 2).

### Cranial Cruciate Ligament Tear

As revealed in Figures 1 and 2, there was no occurrence of CCL in either intact male or intact female dogs, or in late-neutered females. However, in early-neutered dogs, the occurrence reached 5.1 percent in males and 7.7 percent in females, representing significant differences in occurrence from both intact and late-neutered dogs (K-M: p<0.05, Table 4). The mean age of CCL onset in early-neutered males was 3.6 years and the single late-neutered male dog diagnosed with CCL was 7.4 years. The mean age of onset of CCL for early-neutered female dogs was 4.8 years. For CCL, no differences were found between neutered males with and without CCL with regards to their BCS (means 5.8 and 5.8 respectively; CPH: p  = 0.48). Likewise, no differences in mean BCS were found between neutered females with and without CCL (means 5.8 and 5.8 respectively; CHP: *p*  = 0.26).

### Lymphosarcoma

Although the rates of occurrence of this disease were lower in both male and female intact dogs, than in the early-neutered dogs, the difference was statistically significant only in males. Early-neutered males had nearly 3 times the occurrence of LSA as intact males and no cases of LSA were observed in the late-neutered males (K-M: p<0.05, Table 4, Figure 1). The mean ages of LSA onset for intact and early-neutered male dogs were 5.3 and 5.8 years respectively.

### Hemangiosarcoma

Figure 2 reveals that late-neutered females at 7.4 percent were diagnosed with HSA over 4 times more frequently than intact females with 1.6 percent and early-neutered females with 1.8 percent, both significant differences (RR = 6.10, 95% CI  = 1.18, 31.37 and RR = 7.48, 95% CI = 1.79, 31.30). The mean ages of HSA onset for intact, early-neutered, and late-neutered female dogs were 6.4, 7.6, and 3.2 years, respectively. No differences were apparent in males with regard to neutering and the occurrence of HSA (Figure 1).

### Mast Cell Tumor

Figure 2 portrays the findings regarding MCT in female dogs, which did not occur in intact females, but was diagnosed in 2.3 percent of early-neutered females and 5.7 percent of late-neutered females. The RR cannot be estimated when disease occurrence is zero in one comparison group, as in the intact females. However, the wide difference in MCT occurrence between intact and late-neutered females was meaningful, given that the MCT occurrence in late neutered females and early neutered females was significant (RR = 4.46, 95% CI = 1.11, 17.82). The mean ages of MCT onset for the early-neutered and late-neutered female dogs were 6.2 and 6.5 years, respectively. No differences were found in the occurrence of MCT in male Golden Retrievers (Figure 1).

## Discussion

This is the first study of the effects of neutering on an array of joint disorders and cancers in the same breed of dog, using a single database and examining the variables of gender and early and late neutering versus leaving the dogs gonadally intact. No cases of MC were diagnosed in intact females in this study. This finding is partially explained by the relatively low frequency in which MC is diagnosed in Golden Retrievers [16]. While this finding contrasts with the general concern expressed about the risk of MC in gonadally intact females [12], [21], [22], it is consistent with the recent findings from a systematic meta-analysis finding a weak link, if any, between neutering and reduced risk of MC [13].

For all five diseases analyzed in the present study, the disease rates in males and/or females were significantly increased when neutering was performed early and/or late. When a disease occurred in intact dogs, the occurrence was typically one-fourth to one-half that of early- and/or late-neutered dogs. When no intact dogs were diagnosed with a disease, such as with CCL in both sexes and MCT in females, the occurrence in early- and/or late-neutered dogs ranged between 4 and 8 percent of the sample.

The results are consistent with all of the previously reported findings, mentioned in the introduction, of neutering in males and/or females in increasing the likelihood of HSA, LSA, MCT and CCL by about the same degree. However, this is the first study to specifically report an effect of late neutering on MCT and HSA. In the case of HD, which was doubled in the early-neutered males in the present study, the previous study reported a significant increase by only 17 percent in neutered dogs grouped together [15]. These contrasting differences with the effects of neutering on HD profile the value of the approach of the present study in focusing on just one breed and separating out the effects of gender and early versus late neutering.

An important point to make is that the results of this study, being breed-specific, with regard to the effects of early and late neutering cannot be extrapolated to other breeds, or dogs in general. Because of breed-specific vulnerabilities, certain diseases being affected by neutering in Golden Retrievers may not occur in other breeds. By the same token, different joint disorders or cancers may be increased in likelihood in a different breed. A full understanding of the disease conditions affected by neutering across an array of different breeds will require several more breed-specific studies.

A logical question to ask with regard to the joint disorders of HD and CCL is if those neutered dogs diagnosed with the disorder were carrying relatively more weight on their joints, which may have predisposed them to the disorder. Therefore, once an effect of early neutering was found with regard to HD (males) and CCL (males and females), the CPH model was applied to reexamine the effect of early neutering, after adjusting for differences in BCS. While neutering is expected to lead to a greater gain in body weight than in intact dogs [17], [18], the BCS of early-neutered dogs with the disorders and the early neutered comparison groups without the disorders were not significantly different – and, in fact quite similar – indicating that weight on the joint was not a major determinant in the occurrence of these joint disorders. Using the CPH model to compare early-neutered with intact dogs, for both HD and CCL, neither neutering status nor BCS was significant, indicating that the two factors are fairly highly confounded. This implies that the occurrence of HD and CCL in early-neutered dogs is a combined function of the effect of neutering on growth plates, as well as the increase in weight on the joints brought on by neutering. As mentioned, when only early-neutered dogs with and without HD or CCL were compared with respect to their BCS, no differences were found between early-neutered males with and without these joint disorders.

As for the pathophysiological reasons for the joint disorders, one can point to the role of gonadal hormones in controlling the closure of bone growth plates [23], [24]. An atypical growth plate closure, resulting from the absence of gonadal hormones, may increase the chance of a clinically apparent joint disorder, such as HD, CCL, and possibly ED. Confounding factors that may influence the nature of a neuter-related joint disorder are the breed-specific gender vulnerabilities, including growth rate differences, as well as the timing of growth plate closure, which occurs more quickly in males than in females. In the males of this study, the occurrence of HD was doubled in the cases with early androgen removal as compared with intact males, but in females, removal of the ovaries did not appear to be associated with an increased likelihood of HD. This presumably reflects the effect of gender on growth-plate development. However, growth-plate disturbance in both males and females seems to have played a role in the occurrence of CCL in early-neutered dogs. This joint disorder was not diagnosed in either intact males or females. The mean age of CCL onset was later in life than in HD (about 4 years and 2 years, respectively).

The role of gonadal hormone removal in the occurrence of various cancers appears to be more complicated. The effects of early neutering on the increased rate of LSA, especially in males, contrast with the effects of late neutering in females on MCT and HSA. The effects of late neutering associated with the occurrence of MCT and HSA in females bring up the issue of the role of timing of estrogen alteration. One possibility is suggested by the role of estrogen removal and microsatellite instability in colon cancer development in women. Based on clinical findings, it is speculated that estrogen secretion may sensitize the pathways involved in microsatellite instability. While estrogen remains in the system, it is protective against microsatellite instability-positive cancer cell activation and reduces the risk of colon cancer. However, upon estrogen removal, microsatellite instability-positive cancer cells become activated resulting in an increased occurrence of colon cancer [25].

Applying this concept to the role of neutering on HSA and MCT in female dogs, this study suggests that with early neutering, before an estrous period, the cells that could become neoplastic are not sensitized to estrogen and neutering would not affect disease occurrence. However, after exposure to estrogen through several estrous cycles, potentially neoplastic cells could be sensitized, but as long as the female is left intact, the estrogen is protective. Then, if neutered after several estrous cycles, the estrogen-sensitized cells could become neoplastic, hence a higher rate of HSA and especially MCT in late-neutered than early-neutered females. Obviously, much remains to be learned that could be explored with a large database with regard to the specific effects of estrogen in these cancers.

The findings presented here are clinically relevant in two realms. For dog owners and service dogs trainers and caretakers using the popular Golden Retriever as the service dog, the study points to the importance of acquiring information needed for deciding upon if and when to neuter. Specifically for Golden Retrievers, neutering males well beyond puberty should avoid the problems of increased rates of occurrence of HD, CCL, and LSA and should not bring on any major increase in the rates of HSA and MCT (at least before nine years of age). However, the possibility that age-related cognitive decline could be accelerated by neutering should be noted [26]. For females, the timing of neutering is more problematical because early neutering significantly increases the incidence rate of CCL from near zero to almost 8 percent, and late neutering increases the rates of HSA to 4 times that of the 1.6 percent rate for intact females and to 5.7 percent for MCT, which was not diagnosed in intact females.

The findings of this study also have important implications for investigators looking for canine models for research on various forms of cancer [27], [28]. For some cancers of interest, not only may breeds vary in predisposition but also the possibility of interactions between gender, gonadal hormone influences, and timing of gonadal hormone alteration (if any), should be taken into account in selecting the model and in investigating causal factors to be explored.

## References

[1] Trevejo R, Yang M, Lund EM (2011) Epidemiology of surgical castration of dogs and cats in the United States. J Am Vet Med Assoc 238: 898–904. 21453178 10.2460/javma.238.7.898

[2] Sallander M, Hedhammer A, Rundgren M, Lindberg JE (2001) Demographic data of population of insured Swedish dogs measured in a questionnaire study. Acta Vet Scand 71–80. 10.1186/1751-0147-42-71 PMC2202336 11455903

[3] Kubinyi E, Turcsan B, Miklosi A (2009) Dog and owner demographic characteristicsand dog personality trait associations. Behav Processes 81: 392–401. 19520239 10.1016/j.beproc.2009.04.004

[4] Diesel G, Brodbelt D, Laurence C (2010) Survey of veterinary practice policies andopinions on neutering dogs. Vet Rec 166: 455–458. 20382933 10.1136/vr.b4798

[5] Ru G, Terracini B, Glickman LT (1998) Host related risk factors for canine osteosarcoma. Vet J 156: 31–39. 9691849 10.1016/s1090-0233(98)80059-2

[6] Cooley DM, Beranek BC, Schlittler DL, Glickman MW, Glickman LT, et al (2002) Endogenous gonadal hormone exposure and bone sarcoma risk. Cancer Epidemiol Biomarkers Prevent 11: 1434–1440. 12433723

[7] Ware WA, Hopper DL (1999) Cardiac tumors in dogs: 1982–1995. J Vet Intern Med 13: 95–103. 10225598 10.1892/0891-6640(1999)013<0095:ctid>2.3.co;2

[8] Prymak C, McKee LJ, Goldschmidt MH, Glickman LT (1988) Epidemiologic, clinical, pathologic, and prognostic characteristics of splenic hemangiosarcoma and splenic hematoma in dogs: 217 cases (1985). J Am Vet Med Assoc 193: 706–712. 3192450

[9] Villamil JA, Henry CJ, Hahn AW, Bryan JN, Tyler JW, et al (2009) Hormonal and sex impact on the epidemiology of canine lymphoma. J Cancer Epidemiol 2009: 1–7 doi:10.1155/2009/591753. 10.1155/2009/591753 PMC2859020 20445802

[10] Teske E, Naan EC, van Dijk E, Van Garderen E, Schalken JA (2002) Canine prostate carcinoma: epidemiological evidence of an increased risk in castrated dogs. Mol Cell Endocrinol 197: 251–255. 12431819 10.1016/s0303-7207(02)00261-7

[11] White CR, Hohenhaus AE, Kelsey J, Procter-Grey E (2011) Cutaneous MCTs: Associations with spay/neuter status, breed, body size, and phylogenetic cluster. J Am Anim Hosp Assoc 47: 210–216. 21498594 10.5326/JAAHA-MS-5621

[12] Root Kustritz MV (2007) Determining the optimal age for gonadectomy of dogs and cats. J Am Vet Med Assoc 231: 1665–1675. 18052800 10.2460/javma.231.11.1665

[13] Beauvais W, Cardwell JM, Brodbelt DC (2012) The effect of neutering on the risk of mammary tumours in dogs – a systematic review. J Small Anim Pract 53: 314–322. 22647210 10.1111/j.1748-5827.2011.01220.x

[14] Duerr FM, Duncan CG, Savicky RS, Park RD, Egger EL, et al (2007) Risk factors for excessive tibial plateau angle in large-breed dogs with cranial cruciate disease. J Am Vet Med Assoc 231: 1688–1691. 18052804 10.2460/javma.231.11.1688

[15] Witsberger TH, Villamil JA, Schultz LG, Hahn AW, Cook JL (2008) Prevalence of, and risk factors for, hip dysplasia and cranial cruciate ligament deficiency in dogs. J Am Vet Med Assoc 232: 1818–1824. 18598150 10.2460/javma.232.12.1818

[16] Glickman L (1998–1999) Golden Retriever Club of America National Health Survey. Golden Retriever Club of America.

[17] Kasström H (1975) Nutrition, weight gain and development of hip dysplasia. An experimental investigation in growing dogs with special reference to the effect of feeding intensity. Acta Radiologica 344: 135–179, Supplementum. 1066031

[18] Duval JM, Budsberg SC, Flo GL, Sammarco Jl (1999) Breed, sex, and body weight as risk factors for rupture of the cranial cruciate ligament in young dogs. J Am Vet Med Assoc 215: 811–814. 10496133

[19] Kaplan EL, Meier P (1958) Nonparametric estimation from incomplete observations. J Am Stat Assoc 53: 457–481.

[20] Rothman KJ and Greenland S (1998) Modern Epidemiology. Philadelphia: Lippincott Williams & Wilkins.

[21] Egenvall A, Bonnett BN, Ohagen P, Olson P, Hedhamar A, et al (2005) Incidence of, and survival after, mammary tumors in a population of over 80,000 insured female dogs in Sweden from 1995 to 2002. Prevent Vet Med 69: 109–127. 10.1016/j.prevetmed.2005.01.014 15899300

[22] Zatlouka J, Lorenzova J, Tichy F, Necas A, Kecova H, et al (2005) Breed and age as risk factors for canine mammary tumours. Acta Vet Brno 74: 103–109.

[23] Salmeri KR, Bloomberg MS, Scruggs SL, Shille V (1991) Gonadectomy in immature dogs: Effects on skeletal, physical, and behavioral development. J Am Vet Med Assoc 198: 1193–1203. 2045340

[24] Grumbach M (2000) Estrogen, bone growth and sex: a sea of change in conventional wisdom. J Ped Endocrinol Metab 13: 1439–1455. 10.1515/jpem-2000-s619 11202221

[25] Slattery ML, Potter JD, Curtin K, Edwards S, Ma K, et al (2001) Estrogens reduce and withdrawal of estrogens increase risk of microsatellite instability-positive colon cancer. Cancer Res 61: 126–130. 11196149

[26] Hart BL (2001) Effects of gonadectomy on subsequent development of age-related cognitive impairment in dogs. J Am Vet Med Assoc 219: 51–56. 11439769 10.2460/javma.2001.219.51

[27] Vail DM, MacEwen EG (2002) Spontaneously occurring tumors of companion animals as models for human cancer. Cancer Invest 18: 781–792. 10.3109/07357900009012210 11107448

[28] Khanna C, Lindblad-Toh K, Vail D, London C, Bergman P, et al (2006) The dog as a cancer model. Nat Biotechnol 24: 1065–1066. 10.1038/nbt0906-1065b 16964204

